# Effects of Levonorgestrel and progesterone on Oviductal physiology in mammals

**DOI:** 10.1186/s12958-018-0377-3

**Published:** 2018-06-20

**Authors:** Cheng Li, Hui-Yu Zhang, Yan Liang, Wei Xia, Qian Zhu, Duo Zhang, Zhen Huang, Gui-Lin Liang, Rui-Hong Xue, Hang Qi, Xiao-Qing He, Jiang-Jing Yuan, Ya-Jing Tan, He-Feng Huang, Jian Zhang

**Affiliations:** 1 Department of Gynecology, International Peace Maternity and Child Health Hospital, School of Medicine, Shanghai Jiao Tong University, Shanghai, China; 2 Institute of Embryo-Fetal Original Adult Disease Affiliated to Shanghai Jiao Tong University School of Medicine, Shanghai Jiao Tong University, Shanghai, China; 3 Center of Reproductive Medicine, International Peace Maternity and Child Health Hospital, School of Medicine, Shanghai Jiao Tong University, No. 910, Hengshan Rd, Shanghai, 200030 China

**Keywords:** Progesterone, Levonorgestrel, Oviduct, Receptivity, Ciliary beat frequency

## Abstract

**Background:**

Our previous study indicated that emergency contraception, including levonorgestrel and progesterone, could lead to ectopic pregnancy following contraception failure. However, our understanding of the effects of levonorgestrel and progesterone on oviductal physiology is limited.

**Methods:**

The receptivity of the fallopian tubal epithelium after levonorgestrel and progesterone treatment was examined through western blots for receptivity markers and JAr-spheroid-fallopian tubal epithelial cell attachment assays. The ciliary beat frequency was analyzed using an inverted bright-field microscope. Furthermore, an in vivo animal model of embryo-tubal transplantation was also studied to determine the effects of levonorgestrel- and progesterone-induced ciliary beat reduction.

**Results:**

Our results showed that levonorgestrel and progesterone did not change the levels of fallopian tubal epithelial cell receptive markers, including LIF, STAT3, IGFBP1, ITGB3, MUC1, and ACVR1B, or affect JAr-spheroid implantation. However, levonorgestrel and progesterone reduced the ciliary beat frequency in fallopian tubes in a dose-dependent manner. An in vivo model also showed that levonorgestrel and progesterone could lead to embryo retention in the oviducts.

**Conclusions:**

These findings show that levonorgestrel and progesterone can reduce the ciliary beat frequency without altering receptivity, indicating a possible mechanism for progesterone- or levonorgestrel-induced tubal pregnancy.

## Background

Progesterone (P4) plays a crucial role in the regulation of female reproductive physiology [1]. It has been reported that a high level of P4 can interrupt follicular development and thus delay or inhibit ovulation [2]. For this reason, progesterone and its synthetic analogue, levonorgestrel (LNG), are used as contraceptive methods by women of reproductive age.

Levonorgestrel-only pills for emergency contraception (LNG-EC) are available in an over-the-counter form in many countries and can prevent unwanted pregnancies with an efficacy of 52–94% when used within 120 h of unprotected intercourse [3]. Similar to other contraceptive methods, LNG-EC reduces the chance of pregnancy, including both intrauterine pregnancy and occasional ectopic pregnancy (EP); however, cases of EP following LNG-EC failure have been reported in various countries [4, 5]. To confirm the association between EP and LNG-EC, we previously conducted a multi-center case-control study and found that the risk of EP after LNG contraceptive failure was approximately 5-fold higher than that of intrauterine pregnancy [6].

Tubal inflammation, which is typically secondary to genital infection, was generally regarded an important risk factor for EP, but we previously confirmed that tubal pregnancy following LNG-EC failure is associated with lower rates of Chlamydia trachomatis infection, fallopian tubal inflammation, and/or fibrosis compared with general tubal pregnancy [7]. Thus, we hypothesize that LNG, combined with a high progesterone level, might influence fallopian tube physiology rather than tubal morphology or salpingitis.

The transport of embryos in the fallopian tubes is believed to be facilitated through the fallopian tube physiology, which involves ciliary activity and muscular contractions. Previous studies have reported that progesterone can suppress the epithelial ciliary beat frequency in human fallopian tubes by 40–50% [8]. In addition, the administration of progesterone also decreases the contractions of the longitudinal muscular layer of human fallopian tubes compared with the baseline value [9]. Although previous studies have investigated the physiological effects of progesterone on tubal ciliary beats and smooth muscle contractions [8, 9], the effects of the super-physiological P4 levels induced by LNG-EC remain unclear. Although LNG-EC is a synthetic analogue of progesterone, its structure and pharmacological properties, including its effective dose, metabolism, pharmacokinetics, bioavailability and binding to serum binding proteins, differs from those of progesterone. Furthermore, there is no reliable information regarding the influence of LNG on fallopian tubal receptivity, embryo-tubal transportation or implantation. For these reasons, we explored whether LNG, combined with a super-physiological progesterone level, would affect oviduct function, which is involved in the occurrence of EP.

## Methods

### Collection and incubation of human fallopian tubes

After obtaining written consent and approval from the local ethical committee, we collected samples of fallopian tubes at the mid-luteal phase from patients undergoing hysterectomies for benign conditions (uterine leiomyoma)*.* All patients had regular menstrual cycles and had not used any hormonal medication within 3 months. The collected tissues were rinsed several times to remove all visible blood, and the muscularis and serosa were then removed. Pieces of tissue (1–2 mm^2^) were dissected from the ampulla portions of the fallopian tubes and treated with different doses of LNG (Sigma-Aldrich, St Louis, MO, USA) and P4 (Sigma-Aldrich, St Louis, MO, USA) for 24 h in an incubator at 37 °C.

### Cell culture

A human fallopian tubal epithelial cell line (OE-E6/E7) was obtained from Dr. Kai-Fai Lee, University of Hong Kong. The OE-E6/E7 cell line is an immortalized human fallopian tubal epithelium cell line established by the University of Hong Kong, and these cells are characterized by human oviduct-specific glycoproteins, estrogen receptors, and cytokeratin [10]. The OE-E6/E7 cells were cultured at 37 °C in DMEM/F12 culture media (Invitrogen, Paisley, UK) supplemented with 1% penicillin and streptomycin (Invitrogen), 10% fetal bovine serum (Invitrogen) and L-glutamine (Invitrogen) in a 5% CO_2_ atmosphere.

### JAr spheroid-fallopian tubal epithelial cell attachment assays

JAr cells (JAr, HTB-144, ATCC, Manassas, VA, USA) are a trophoblastic tumor cell line of placental origin that express the placental hormone and differentiate into syncytiotrophoblasts. We used multicellular spheroids of human choriocarcinoma JAr cells as an in vitro attachment model as previously described [11]. We treated OE-E6/E7 cells at 40–50% confluency with 10 nmol/L 17β-estradiol (Sigma-Aldrich, St Louis, MO, USA) and 1 nmol/L P4 (Sigma-Aldrich, St Louis, MO, USA) to mimic the hormonal environment under normal physiological conditions. The cells were treated with LNG and P4 at various concentrations until full confluency was reached. At 80–90% confluency, the OE-E6/E7 cells were treated with various concentrations of LNG and P4 for 24 h, and JAr spheroids were transferred onto the surface of a confluent monolayer of OE-E6/E7 cells. The cultures were maintained in the culture medium for 6 h. Non-adherent spheroids were removed by centrifugation of the cell culture plates with the cell surface facing down at 15 g for 10 min. We counted the attached spheroids under a light microscope, and the results are expressed as percentages of the total number of seeded spheroids (% adhesion).

### Measurement of the ciliary beat frequency

The ciliary beat frequency (CBF) was measured at 37 °C under an inverted bright-field microscope (Nikon TE2000, Nikon Instruments, Inc., Melville, NY, USA). Video sequences of the moving cilia were acquired with a 12-bit high-speed camera (Prosilica EC1020, Prosilica Inc., Burnaby, Canada) at a rate of 30 frames per s for 10 s. The CBF was calculated using ciliaFA software, a plugin for ImageJ (software version 1.49 t; NIH, USA) that extracts pixel intensities and performs fast Fourier transformation using Microsoft Excel (2016 professional edition; Microsoft Corporation, WA, USA) [12].

### Quantitative real time-PCR (qRT-PCR) analyses

The total RNA from scraped cells was extracted using the RNAiso reagent (TAKARA, Dalian, China) and then reverse-transcribed according the manufacturer’s instructions (TAKARA, Dalian, China). qRT-PCR was performed with a QuantStudio™ 7 flex system (Applied Biosystems, Foster City, CA, USA) using the primer sequences listed in Table 1. The threshold cycles were determined, and relative gene expression levels were calculated using the 2^-ΔΔCT^ method with glyceraldehyde-3-phosphate dehydrogenase as the endogenous control.

**Table 1:** Primer sequences of PCR

Gene	Forward primer	Reverse primer
18 s	GTAACCCGTTGAACCCCATT	CCATCCAATCGGTAGTAGCG
STAT3	CAGTGACAGCTTCCCAATGG	ACTGCTGGTCAATCTCTCCC
IGFBP1	TGATGGCCCCTTCTGAAGAG	TCTCCTGTGCCTTGGCTAAA
ITGB3	TGACGAAAATACCTGCAACCG	GCATCCTTGCCAGTGTCCTTAA
MUC1	GAAAGAACTACGGGCAGCTG	GCCACCATTACCTGCAGAAA
LIF	TGAACCAGATCAGGAGCCAA	GACTATGCGGTACAGCTCCA
ACVR1B	AAAGACAAGACGCTCCAGGA	ATACTTCCCCAAACCGACCC

### Western blotting

OE-E6/E7 cells were lysed in RIPA buffer supplemented with a protease inhibitor cocktail (Millipore, Darmstadt, Germany). Protein loading was normalized using the total protein concentrations determined through Bradford assays. Samples (30 μg/lane) were separated on a 12% sodium dodecyl sulfate-polyacrylamide gel and transferred onto Protran Immun-Blot nitrocellulose transfer membranes (Schleicher & Schuell Bioscience GmbH, Dassel, Germany). Antibodies against β-actin (1:5000, Proteintech, IL, USA), IGFBP1 (1:1000, Proteintech, IL, USA), ITGB3 (1:1000, Proteintech, IL, USA), MUC1 (1:1000, Proteintech, IL, USA), ACVR1B (1:1000, Proteintech, IL, USA) and STAT3 (1:1000, Cell Signaling Technology, Danvers, MA, USA) were used as primary antibodies, and horseradish peroxidase-conjugated goat anti-rabbit IgG (1:5000, Cell Signaling Technology, Danvers, MA, USA) and goat anti-mouse IgG (1:5000, Cell Signaling Technology, Danvers, MA, USA) were used as secondary antibodies. Specific signals were visualized by the enhanced chemiluminescence method as previously described [13].

### Animal experiments and embryo-tube transportation assays

Eight-week-old C57BL/6 J mice were used in this study (Shanghai Research Center for Model Organisms). All animal experiments were approved by the Medical Ethics Committee of Shanghai Research Center for Model Organisms. The mice were housed in a room at 25 °C with a 12-h light:12-hdark cycle and 50–60% humidity and were given a standard diet (containing 10% fat) and water. Female mice (6–8 weeks of age) were mated randomly, and vaginal plug-positive mice were immediately injected intraperitoneally with saline, LNG (8 mg/kg), or P4 (8 mg/kg). Twelve hours after observation of the vaginal plug, the mice were sacrificed via cervical dislocation to measure the CBF. Embryo-tube transportation assays were conducted as described by Ning et al. [14]. Seventy-four hours after observation of the vaginal plug, the oviducts and uterus were ligated. The embryos were flushed from the oviducts or uteri with PBS. We counted the embryos remaining in the oviducts, and the results are expressed as percentages of the total number of embryos.

### Statistical analysis

All the results are expressed as the means ± standard deviation. To determine the statistical significance of the differences among the treatments, one-way analysis of variance and Tukey-Kramer multiple comparisons tests were performed to compare the relative efficacy of each treatment (PRISM software version 6.0; GraphPad). A probability of *p* < 0.05 was considered to indicate a significant difference.

## Results

### Effect of LNG on fallopian tube epithelium receptivity

**Fig. 1:**
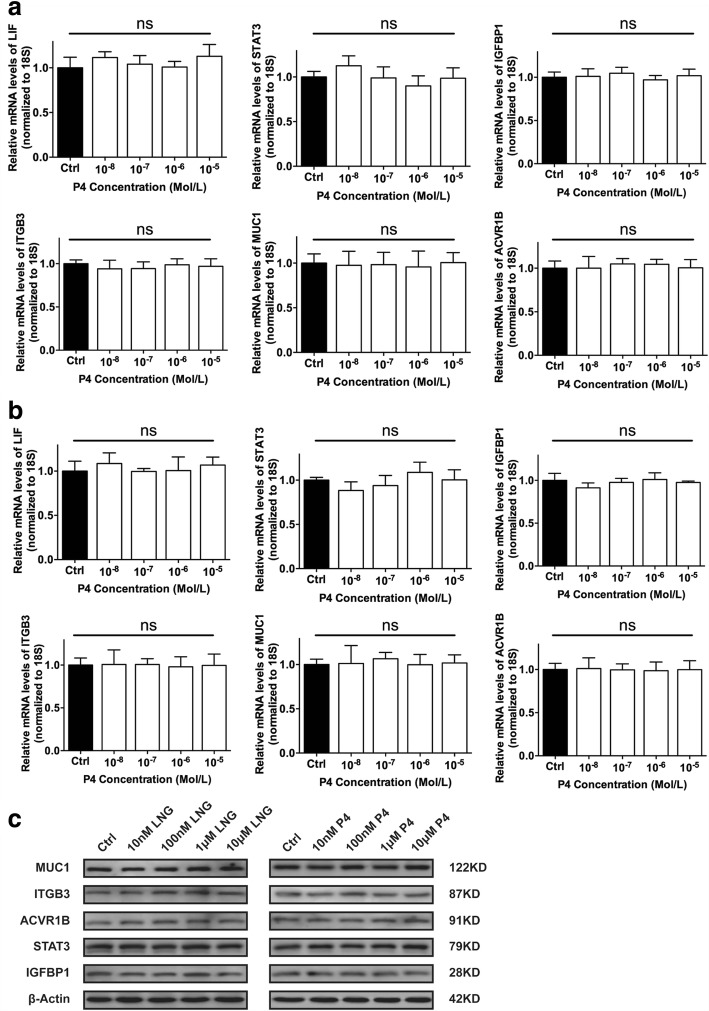
Effects of different concentrations of LNG and P4 on the expression of receptivity markers in the fallopian tubes. **a-b** mRNA expression levels of LIF, STAT3, IGFBP1, ITGB3, MUC1, and ACVR1B in OE-E6/E7 cells following treatment with different concentrations of LNG and P4 (*n* = 3 in each group; ns, not significant); **c** protein expression levels of MUC1, ITGB3, ACVR1B, STAT3, and IGFBP1 in OE-E6/E7 cells following treatment with different concentrations of LNG and P4

**Fig. 2:**
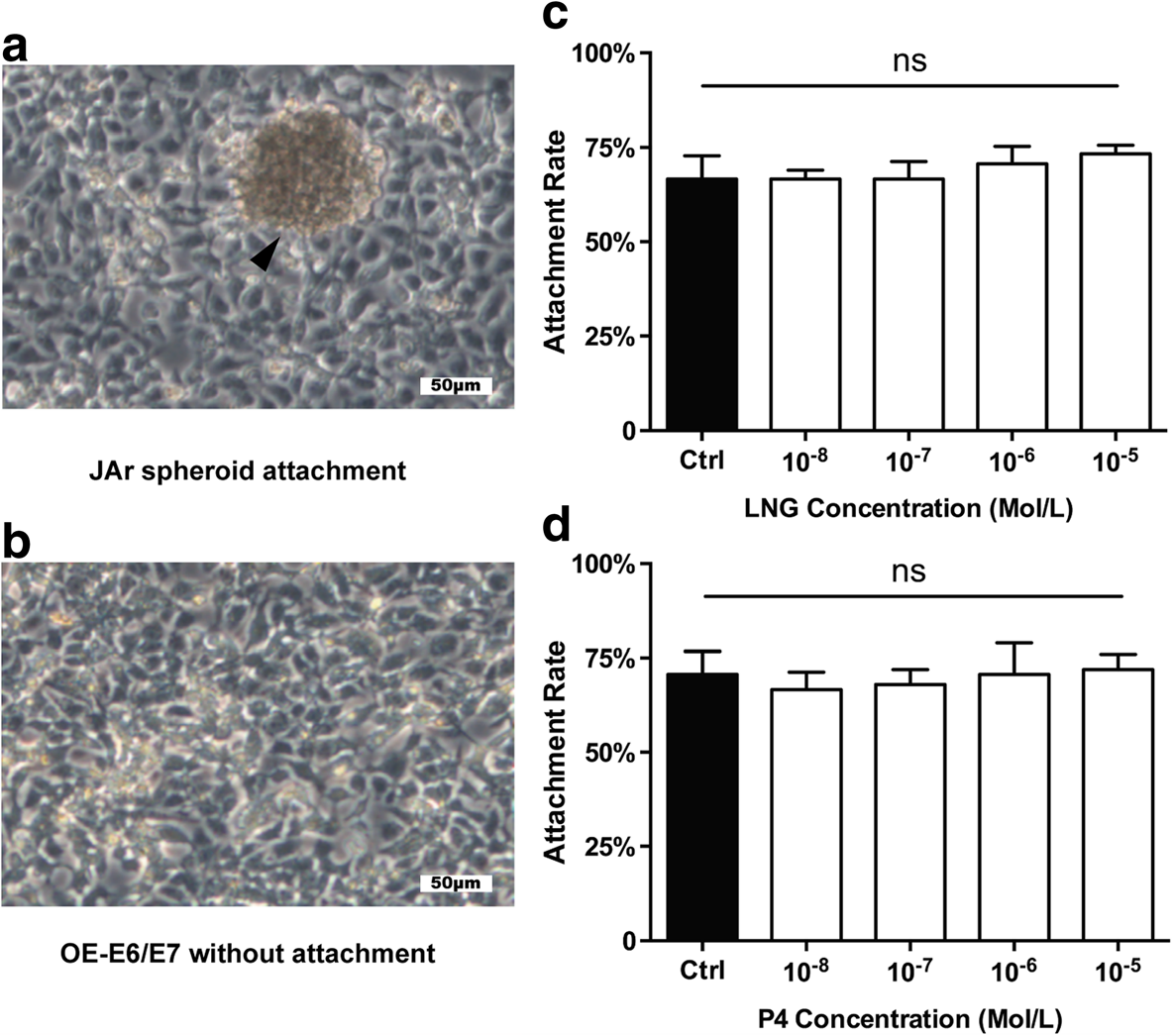
Effects of different concentrations of LNG and P4 on JAr spheroid- fallopian tubal attachment rates. **a** JAr spheroids were selected (arrow) and attached to OE-E6/E7 monolayers; **b** OE-E6/E7 monolayer without attached JAr spheroids; **c-d** rates of the attachment of JAr spheroids to OE-E6/E7 cells treated with different concentrations of LNG and P4 (*n* = 3 in each group; ns, not significant)

To determine whether LNG affects the receptivity of the fallopian tubal epithelium, we subjected the tubal epithelial cell line OE-E6/E7 to different doses of LNG and P4 and detected the expression of various receptive markers, including LIF, STAT3, IGFBP1, ITGB3, MUC1, and ACVR1B, through qRT-PCR and western blot analyses. However, the expression of these receptive markers did not show any significant changes following the administration of LNG or P4, regardless of the dose (Fig. 1). Furthermore, we performed JAr spheroid-fallopian tubal epithelial cell attachment assays. Spheroids of approximately 60–150 μm were produced from JAr cells and allowed to attach to a monolayer of OE-E6/E7 cells (Fig. 2a) that had been previously treated with different doses of LNG and P4, and the percentages of attached JAr spheroids did not show any significant differences among the groups (Fig. 2c-d).

### Effect of different concentrations of LNG on the CBF

**Fig. 3:**
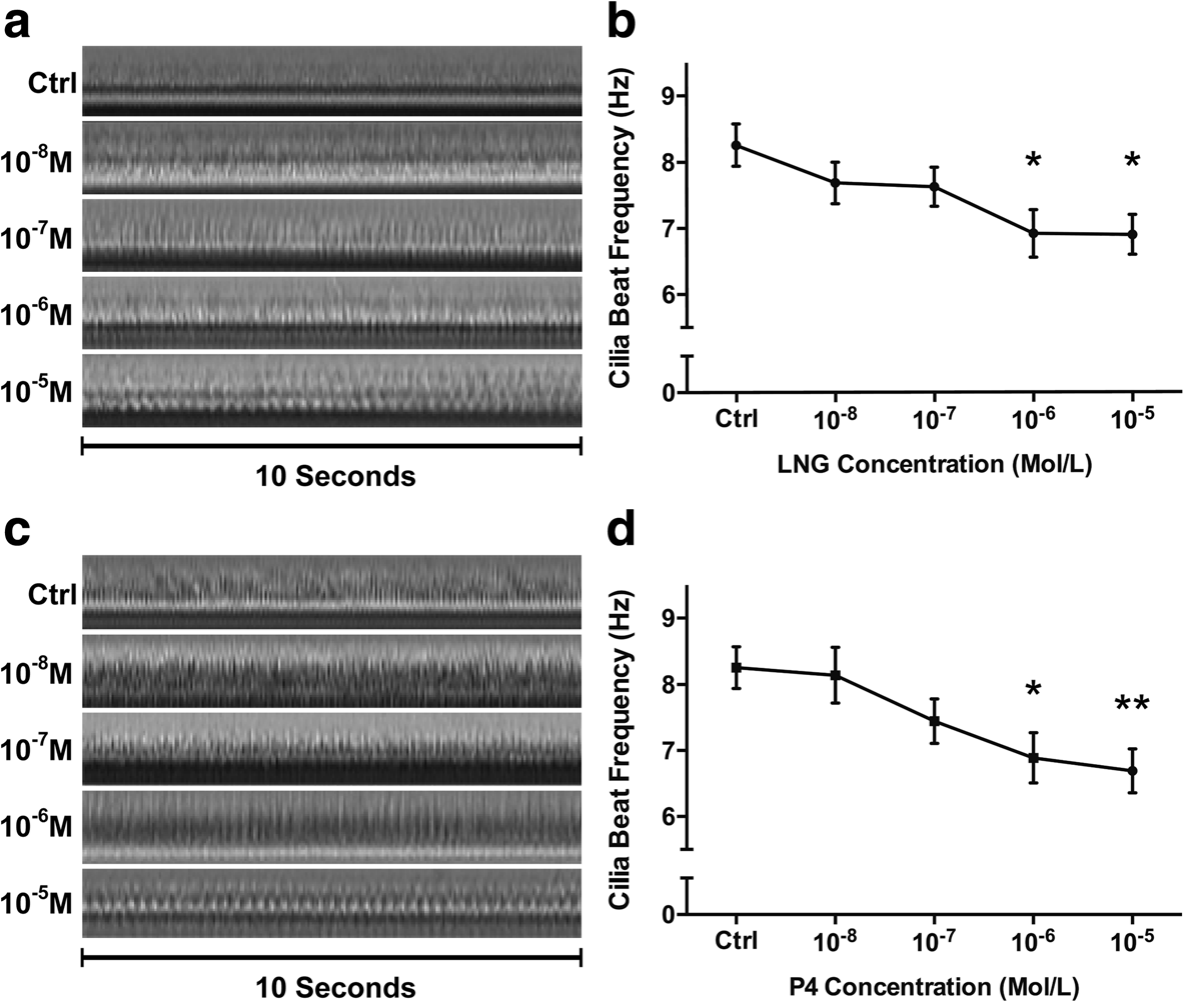
Effects of different concentrations of LNG and P4 on the tubal CBF in vitro. **a** Orthographic views of the ciliary beat frequency following treatment with different concentrations of LNG (one ciliary beat represents one shift from bright to dark on the timeline); **b** LNG decreased the CBF in vitro in a dose-dependent manner (*n* = 8 in each group; *, *p* < 0.05); **c** orthographic views of the ciliary beat frequency following treatment with different concentrations of P4; **d** P4 decreased the CBF in vitro in a dose-dependent manner (*n* = 8 in each group; *, *p* < 0.05; **, *p* < 0.01)

To determine whether LNG, together with altered P4 levels, had a dose-dependent effect on the tubal CBF, we cultured tubal epithelial explants with LNG and P4 at doses ranging from 10^− 8^ to 10^− 5^ mol/L. The CBF decreased with increases in the LNG concentration (Fig. 3a-b). The CBF of the explants incubated with LNG at a concentration of 10^− 6^ mol/L (6.92 ± 0.36 Hz) and 10^− 5^ mol/L (6.89 ± 0.30 Hz) was decreased significantly compared with that of explants in the control medium (8.23 ± 0.32 Hz). Similar results were obtained with the P4 treatments. Treatment with P4 at 10^− 6^ mol/L and 10^− 5^ mol/L decreased the CBF to 6.89 ± 0.38 Hz and 6.69 ± 0.33 Hz, respectively, from 8.26 ± 0.32 Hz, which was the value obtained for the explants in the control medium (Fig. 3c-d).

**Fig. 4:**
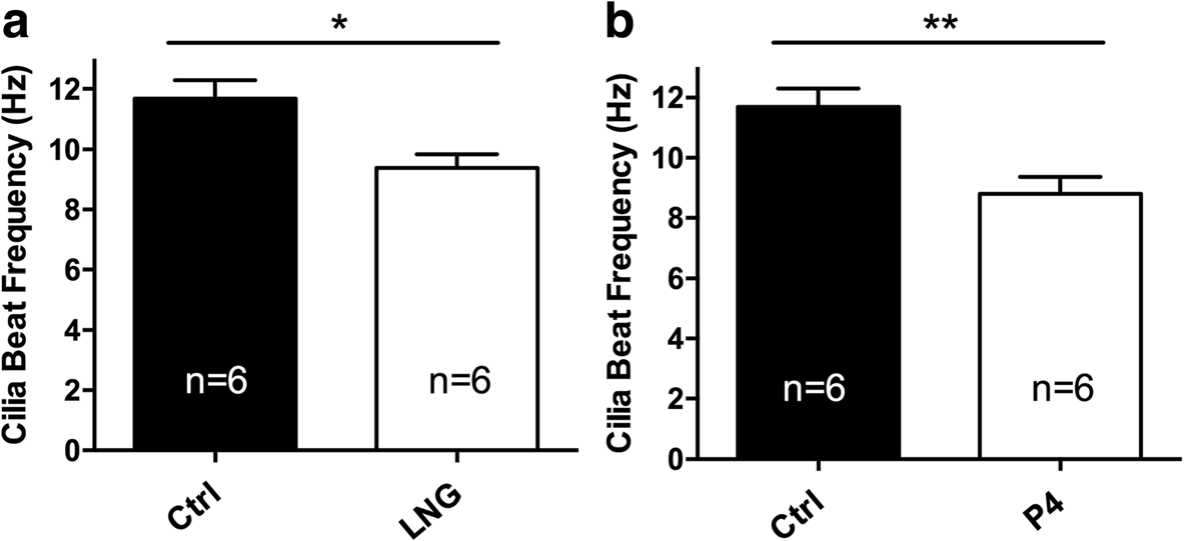
Effects of LNG and P4 on the tubal CBF in mice. **a** LNG decreased the tubal CBF in mice (*n* = 6 in each group; 9.39 ± 0.45 Hz vs. 11.69 ± 0.60 Hz, *p* = 0.012); **b** P4 decreased the tubal CBF in mice (*n* = 6 in each group; 8.80 ± 0.56 Hz vs. 11.69 ± 0.60 Hz, *p* = 0.006)

To further confirm these findings in vivo, we analyzed the CBF of C57BL6/J mice after an intraperitoneal injection of saline, LNG (8 mg/kg), or P4 (8 mg/kg). The in vivo CBF of mice was significantly decreased after treatment with LNG (9.39 ± 0.45 Hz vs. 11.69 ± 0.60 Hz, *p* = 0.012) or P4 (8.80 ± 0.56 Hz vs. 11.69 ± 0.60 Hz, *p* = 0.006) compared with that of the saline-treated control mice (Fig. 4).

### Effect of LNG on embryo-tube transportation in mice

**Fig. 5:**
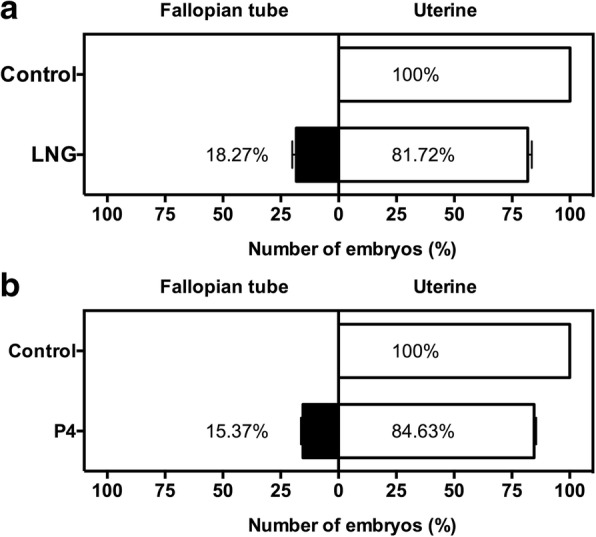
Effects of LNG and P4 on embryo-tubal transportation in mice. **a** An embryo recovery of 18.27% was obtained from the oviducts following the administration of LNG (*n* = 5 in each group); **b** 15.37% of embryos were recovered from the oviducts following the administration of P4 (*n* = 5 in each group)

Because the ciliary beat in the fallopian tubes plays a critical role in embryo transport, we further observed the effects of LNG and the super-physiological level of P4 on embryo transport through the fallopian tube. We counted the percentages of embryos retained in the fallopian tubes in each group and found that all the mice in the LNG group experienced embryo-tube retention, with an average percentage of embryo retention of 18.27%, whereas none of the mice in the saline-treated group experienced embryo-tube retention (Fig. 5a). Consistently, the same effect was also observed in the P4 group, which had an average percentage of embryo retention of 15.37%, compared with the control group, which had an average percentage of 0% (Fig. 5b).

## Discussion

Our findings show that LNG had no effect on fallopian tubal receptivity but revealed a dose-dependent effect on ciliary motility: increases in the concentration of LNG resulted in decreases in the tubal ciliary beat frequency, which ultimately led to embryo retention in the fallopian tubes of mice. This phenomenon might account for the clinical findings of an increased risk of EP following LNG contraceptive failure observed in our previous study [6].

With regard to the mechanism of EP, it is believed that an altered tubal environment and impaired embryo-tubal transportation allow implantation of an embryo in the fallopian tube [15]. Thus, we further explored the effects of LNG, together with a super-physiological P4 level, on both of these physiological functions.

Successful implantation requires a receptive endometrium that is appropriately primed with estrogen and progesterone. In an EP, alterations to the fallopian tubal receptivity might be a response to the sequence of factors or cytokines that alter various physiological functions, including the promotion of embryo implantation [15]. However, no previous studies have that indicated whether LNG, together with a super-physiological P4 level, can change the secretion of implantation factors that induce apposition, adhesion and invasion in fallopian tubal epithelium cells to promote embryo-tubal implantation. We thus detected the expression of LIF, MUC1, ITGB3, ACVR1B, STAT3, and IGFBP1, which are known implantation factors, in the human fallopian tubal cell line OE-E6/E7 after treatment with different does of LNG and P4. The results suggest that LNG and P4 have no effects on the receptivity of fallopian tubal cells by stimulating secretion or altering the expression levels of receptive factors. The results also confirm the findings of our in vitro JAr spheroid-fallopian tubal epithelial cell attachment assays, which showed that LNG, together with a super-physiological P4 level, do not affect fallopian tubal receptivity. However, an in vivo model for tubal pregnancy in rodents has not been established because the abdominal cavity is the most frequent extra-uterine implantation site and only a few cases of tubal pregnancy in primates have been reported to date [16]. Therefore, the in vivo evaluation of tubal implantation is difficult.

Embryo transport through the fallopian tube is managed mainly by the ciliary beat frequency and muscular contractions. Almost 80% of EPs occur in the ampulla, which is characterized by a thin smooth muscle layer and long longitudinal mucosal folds with a high percentage of ciliated cells in the epithelium. However, the isthmus has a thick smooth muscle layer and only one-fourth the number of ciliated cells found in the ampulla [17]. Halbert et al. confirmed that the rate of ovum transport remains unchanged after smooth muscle activity is blocked with isoproterenol, which indicated that ciliary motility was capable of transporting embryos in the absence of muscle contraction [18]. Thus, this study focused on the effect of LNG on the tubal CBF and showed that LNG and P4 decreased the tubal CBF in a dose-dependent manner. Furthermore, our in vivo experiment revealed embryo retention in the fallopian tubes of mice following LNG and P4 administration. However, LNG cannot prevent pregnancies if it is taken after the luteinizing hormone level has begun to increase and ovulation has occurred [2]. At this point, the fertilized ovum would be transported slowly due to the LNG-induced decrease in the CBF. This slow transport will increase the risk of tubal implantation, as reported in our previous study [6]. Because of these findings, we believe that high levels of LNG or P4 decrease the tubal CBF and might contribute to the occurrence of tubal pregnancy following LNG-EC failure.

In addition to the clinical findings of a correlation between EP and contraceptive doses of LNG, the association between high levels of P4 and EP might also provide insight into the reason for the increased EP rates found among women using assisted reproductive technology. Our previous epidemiology study revealed that the risk of EP is significantly increased among women who underwent assisted reproductive technology treatment [19]. Several studies have also indicated that the risk of EP is closely related to fresh embryo transfer [20] and controlled ovarian stimulation (COH) [21]. Notably, transferred embryos are more likely to move from their original transfer position to the fallopian tubes due to the cervix-to-fundus direction of uterine peristalsis following COH [22, 23]. Moreover, the supraphysiological hormone levels induced by COH, particularly elevated P4 levels, might reduce ciliary motility and lead to subsequent embryo retention in the fallopian tube. This finding could also help explain the lower rate of EP in embryo transfer cycles without ovarian hyperstimulation, such as frozen embryo transfer, compared with that observed with fresh embryo transfer. Extension of the clinical implications of the present findings indicates that an increased P4 level in the tubal-uterine environment contributes to ectopic implantation after embryo transfer.

## Conclusion

In summary, our data show that LNG, combined with a super-physiological level of progesterone, can decrease the tubal ciliary beat frequency and thus lead to embryo retention in the fallopian tube without changing tubal receptivity. A better understanding of the role of LNG in modulating the physiology of the human fallopian tube might help delineate the underlying mechanism leading to tubal pregnancies and better prevent tubal pregnancies in the future.

## Abbreviations

ACVR1B: Activin A receptor type 1B
CBF: Ciliary beat frequency
IGFBP1: Insulin-like growth factor binding protein 1
ITGB3: Integrin subunit beta 3
LIF: Leukemia inhibitory factor
LNG: Levonorgestrel
LNG-EC: Levonorgestrel emergency contraception
MUC1: Mucin 1
P4: Progesterone
qRT-PCR: Quantitative real-time polymerase chain reaction
STAT3: Signal transducer and activator of transcription 3
